# Release from Above- and Belowground Insect Herbivory Mediates Invasion Dynamics and Impact of an Exotic Plant

**DOI:** 10.3390/plants8120544

**Published:** 2019-11-26

**Authors:** Lotte Korell, Martin Schädler, Roland Brandl, Susanne Schreiter, Harald Auge

**Affiliations:** 1 Plant Ecology and Geobotany, Department of Ecology, University of Marburg, Karl-von-Frisch-Str. 8, 35032 Marburg, Germany; 2 Institute of Biology, Martin Luther University Halle-Wittenberg, Am Kirchtor 1, 06108 Halle (Saale), Germany; 3 Department of Community Ecology, Helmholtz-Centre for Environmental Research -UFZ, Theodor-Lieser-Str. 4, 06120 Halle, Germany; martin.schaedler@ufz.de (M.S.); harald.auge@ufz.de (H.A.); 4 German Centre for Integrative Biodiversity Research (iDiv), Halle-Jena-Leipzig, Deutscher Platz 5e, 04103 Leipzig, Germany; 5 Animal Ecology, Department of Ecology, University of Marburg, Karl-von-Frisch-Str. 8, 35032 Marburg, Germany; brandlr@biologie.uni-marburg.de; 6 Department of Soil System Science, Helmholtz-Centre for Environmental Research - UFZ, Theodor-Lieser-Str. 4, 06120 Halle, Germany; susanne.schreiter@ufz.de

**Keywords:** biological invasions, diversity, ecosystem functions, enemy release, herbivorous insects, long-term experiment, plant communities, productivity, trophic interactions

## Abstract

The enemy-release hypothesis is one of the most popular but also most discussed hypotheses to explain invasion success. However, there is a lack of explicit, experimental tests of predictions of the enemy-release hypothesis (ERH), particularly regarding the effects of above- and belowground herbivory. Long-term studies investigating the relative effect of herbivores on invasive vs. native plant species within a community are still lacking. Here, we report on a long-term field experiment in an old-field community, invaded by *Solidago canadensis* s. l., with exclusion of above- and belowground insect herbivores. We monitored population dynamics of the invader and changes in the diversity and functioning of the plant community across eight years. Above- and belowground insects favoured the establishment of the invasive plant species and thereby increased biomass and decreased diversity of the plant community. Effects of invertebrate herbivores on population dynamics of *S. canadensis* appeared after six years and increased over time, suggesting that long-term studies are needed to understand invasion dynamics and consequences for plant community structure. We suggest that the release from co-evolved trophic linkages is of importance not only for the effect of invasive species on ecosystems, but also for the functioning of novel species assemblages arising from climate change.

## 1. Introduction

Plant antagonists such as insect herbivores can exert strong effects on assembly processes in plant communities, and on functional aspects of the ecosystem. For instance, insect herbivores are known to mediate plant species co-existence by suppressing dominant plant species, thereby increasing plant species richness [1,2,3] and evenness [4,5]. This effect can be attributed to equalizing or stabilizing mechanisms [6]: Based on a competition–defense trade-off, herbivores can decrease fitness differences among plant species by attacking competitively superior species [7] or they can stabilize coexistence by negative frequency-dependent effects on plant fitness [8,9]. In addition, the negative effect of insect herbivory on fast-growing, competitive plant species is often reflected by a decrease in aboveground biomass of herbaceous plant communities [3,10]. On the other hand, insect herbivores may also increase (or reverse) fitness differences among plant species, thereby weakening co-existence [11] and facilitating succession [1].

Furthermore, insect herbivory is suggested to contribute to invasion success of exotic plant species: According to the enemy-release hypothesis (ERH), invasion success can be attributed to release from co-evolved enemies present in the native range and reduced attack by evolutionary naïve enemies in the invaded range [12,13,14]. This should result in a competitive advantage of invasive species over native species that are still under control by their co-evolved natural enemies, or in terms of coexistence theory, natural enemies reduce the fitness of resident species, thereby promoting invasion [15]. Hence, an explicit test of the ERH requires a biogeographic comparison between the invasive and the native range of the focal plant species [16], and an experimental removal of natural enemies, e.g., insect herbivores, from the invasive species and their native competitors in both regions [12].

Empirical evidence for the ERH is equivocal [17,18,19,20]. Most studies compared herbivore load or herbivore damage among the native and the invasive ranges, or among co-occurring or phylogenetically related native-invasive species pairs [17]. However, differences in herbivore damage or herbivore load do not necessarily translate into changes of individual fitness and, in turn, of population abundance [21,22,23]. Hence, direct experimental tests of the prediction of ERH of how herbivory affects population dynamics of invaders are still rare [24]. In addition, the few insect exclusion experiments testing these predictions usually removed insect herbivores from the invasive plant species (e.g., [25,26]) but not from its native competitors.

Invasions by exotic plant species are among of the most serious threats to biodiversity and are known to drive fundamental changes of biotic interactions, ecosystem processes, and functions [27,28,29]. In communities composed of native species, the loss of biodiversity is commonly accompanied by a loss of primary production [30,31,32]. However, in contrast to this well-studied biodiversity–productivity relationship, invasions are frequently accompanied by an increase of net primary production of the recipient plant community [33,34]. One explanation for this increase in productivity and concomitant decrease in diversity associated with exotic plant invasions may be the dislocation of exotic species from co-evolved relationships, such as those with herbivores [35,36,37]. Species interactions like niche partitioning and facilitation stabilize diversity in co-evolved native plant communities but such effects seem to be less important in assemblages dominated by exotic species [38,39]. According to theory, dominance of exotic species, and hence their impact on the invaded community, should critically depend on the fitness advantage of the exotic invader over resident species [40]. Hereby, we refer to the fitness concept by Chesson [41] in the framework of co-existence theory stating that the fitness of a species “is a summary ability of the species to succeed in the given environment relative to other species in the same guild”. Explicitly testing the ERH thus requires removing herbivores from the whole plant community (including native and invasive species) and investigating the effects on the invasion success of exotic plants. Finally, as exotic species often possess traits that are associated with high productivity [42], a disproportional attack of herbivores on native vs. exotic species could ultimately lead to an increase in the productivity of the invaded community when herbivores are present.

Local abundance of invaders has been proven to be a good indicator of invader impact on native plant abundance and species richness [43,44]. However, herbivore driven shifts in plant abundance can take some years to appear and change over time [3]. So far, very few studies concentrated on shifts in the abundance of invasive and co-occurring native species as a result of differences in herbivory [45,46,47,48,49] and these studies were mainly short- to mid-term experiments (see [50,51]). Long-term herbivore exclusion experiments can provide important insights into how herbivores control population dynamics of invaders and what consequences arise for species richness and productivity at the community level. Moreover, there is increasing evidence that belowground herbivores are equally or even more important in structuring plant communities as aboveground herbivores [1]. However, there are still few studies on effects of belowground herbivores for biological invasions (see review by [52]).

Considering the release of invasive plants from their enemies, we hypothesize that the functional role of above- and belowground insect herbivory in communities invaded by exotic plants will be strikingly different from their role in communities composed of native plant species. Selective feeding of herbivores on native plants, thereby increasing the fitness difference to exotic species, should facilitate the local loss of native plant diversity. Moreover, this process should ultimately also transform plant communities into assemblages dominated by highly productive invasive species, thereby increasing primary production but decreasing diversity. To test these hypotheses, we re-evaluated previously unpublished data from a long-term insect exclusion experiment (see [1]) originally designed to investigate the role of insect herbivory on dynamics of an old-field plant community. During eight years of the experiment, *S. canadensis* sensu lato started to invade experimental plots. *S. canadensis* s. l. is invasive in many parts of the world and is seen as an important threat to European landscapes [53]. Insect exclusion experiments revealed that population dynamics of *Solidago* species in their native range North America is controlled by specialized insect herbivores [4,54,55]. Furthermore, diversity and abundance of the insect herbivore fauna on *S. canadensis* is much lower in Europe compared to the native range, indicating strong release from specialist enemies [56]. Hence, together with these data, our experiment provided a unique opportunity to explicitly test predictions of ERH, as well as to study the impact of this plant invasion on the resident plant community.

## 2. Results

### 2.1. Effect of Insect Herbivory on Population Dynamics of the Invasive Species S. canadensis

**Figure 1:**
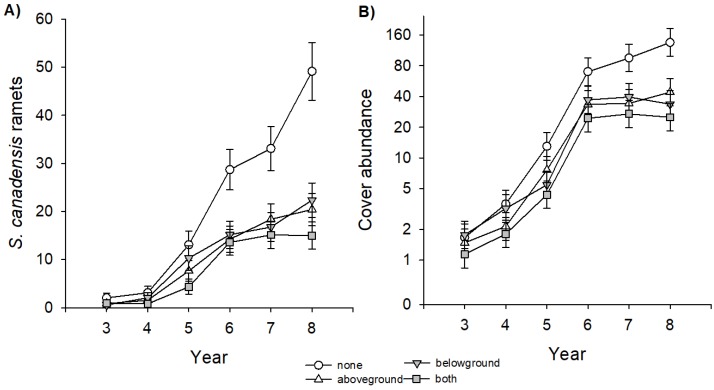
Effects of insect exclusion (no insect exclusion (none), aboveground exclusion (aboveground), belowground exclusion (belowground), and above- and belowground exclusion (both) on the invasive plant species *Solidago canadensis* in a Central European old-field community during eight years of succession. (**A**) Dynamics of the number of ramets and (**B**) of the cover abundance. Data shown are least square means of repeated-measure linear mixed models ± SE. Please note in (**B**), the y-axes is log-scaled. Number of observations = 544 (number of ramets), 480 (cover abundance).

**Figure 2:**
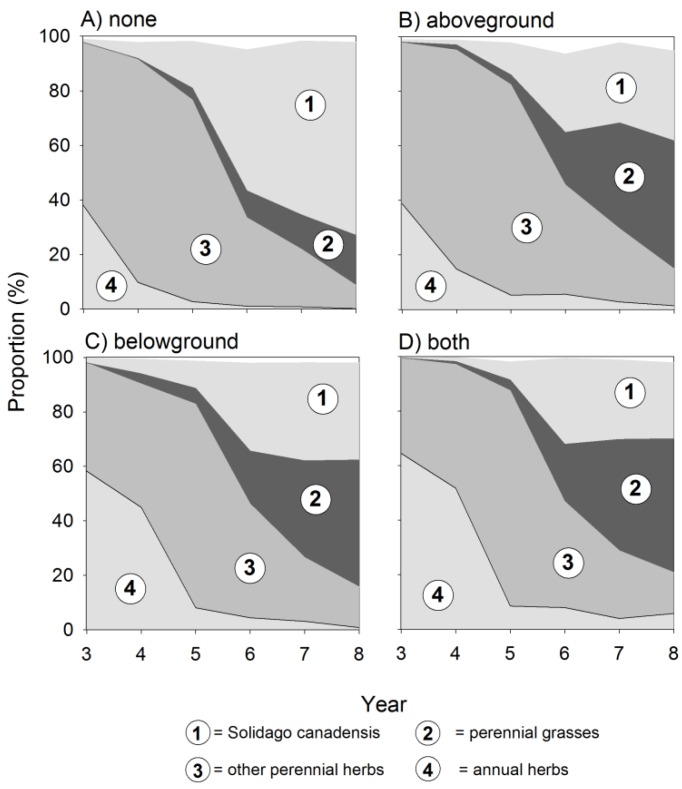
Effects of insect exclusion on the composition of a Central European old-field community during eight years of succession. Dynamics of the community composition with (**A**) no insect exclusion (none), (**B**) aboveground exclusion (aboveground), (**C**) belowground exclusion (belowground), and (**D**) above- and belowground exclusion (both) on the relative abundance on *S. canadensis* and perennial grasses, perennial herbs, and annual herbs. Number of observations = 480.

In our experiment, *S. canadensis* started to establish in the third year of succession, but on plots where insects were excluded, goldenrod reached its final density already in year 6 (Figure 1). This effect was similar on plots where above- and belowground insect herbivores were excluded, and there was no interaction between these two factors. In contrast, on plots with no insect exclusion, abundance of *S. canadensis* increased until the end of our experiment leading to significant differences between treatments. This effect caused a more than tripled number of ramets (Figure 1A; Table 1) and a more than doubled cover abundance (Figure 1B; Table 1). As a consequence, at the end of the experiment, *S. canadensis* dominated the plant community only on plots with no insect exclusion with a mean relative abundance of 71% compared to plots with insect exclusion where the species did not reach values higher than 40% (Figure 2A, Table 2). The exclusion of aboveground insect herbivores increased the cover abundance of native perennial grasses (*F*_1,67_ = 5.6 *) and annual herbs (*F*_1,67_ = 6.1 *), whereas belowground insect herbivores particularly increased the cover abundance of annual herbs (*F*_1,67_ = 12.70 ***), perennial grasses (*F*_1,67_ = 5.57 *), but also of perennial herbs (*F*_1,67_ = 6.0 *).

### 2.2. Consequences for Plant Community Diversity and Biomass

**Figure 3:**
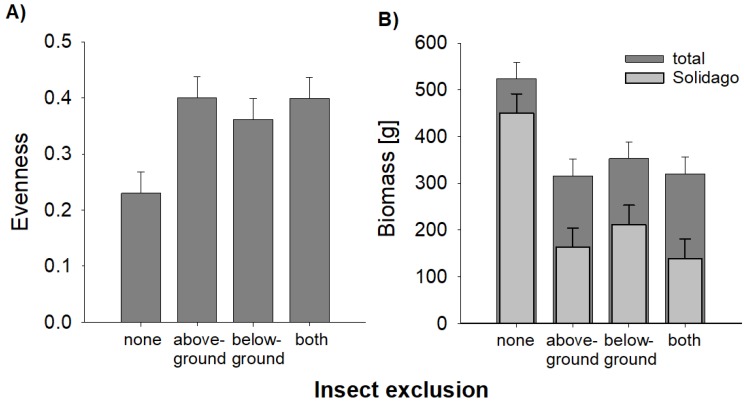
Effects of insect exclusion on evenness and biomass of an old-field community after eight years of succession. (**A**) Evenness based on biomass data, (**B**) total aboveground biomass of vegetation and aboveground biomass of the invasive *Solidago canadensis* on plots with no insect exclusion (none), aboveground insect exclusion (aboveground), belowground insect exclusion (belowground), and with above- and belowground insect exclusion (both). Bars represent the mean and error bars represent one common standard error. Number of observations = 96.

At the end of the experiment, the evenness and species richness (mean ± se: 8.6 ± 0.5) of the plant community were notably lower on plots with no insect exclusion, i.e., control plots with natural insect herbivory (Figure 3), whereas especially the exclusion of insects aboveground led to higher species richness (9.6 ± 0.5) and more evenly distributed abundances of those plant species (Figure 3A, Table 3). This effect was associated with a high dominance of *S. canadensis* on plots with no insect exclusion (Figure 2A, Table 2), as evenness was negatively correlated with the biomass of *S. canadensis* across plots (*R* = −0.53, *p* < 0.001). Moreover, total aboveground biomass of plants was considerably higher on plots with no insect exclusion (Figure 3B; Table 3). Again, this was a consequence of the dominance of *S. canadensis* since the biomass of this species was much larger on control plots (Figure 3B; Table 3).

## 3. Discussion

The enemy-release hypothesis is one of the most cited but also most critically discussed hypotheses in invasion biology [20,57]. However, there is still a lack of long-term experiments to explicitly test the predictions of ERH, and to detect consequences of enemy release on the abundance and species richness of native competitors at the community level. Our results illustrate the importance of such long-term studies, as the effects of invertebrate herbivores on invader establishment and resulting changes in native community structure first appeared after six years and became stronger over time (see also [3]). Our study revealed an association between the decline in biodiversity of native species and the release of a highly invasive species (*Solidago canadensis*) from competition. We suggest this relationship to be mediated by disproportional above- and belowground insect herbivory on native relative to the invasive species.

As proposed by Keane and Crawley [12] we found stronger negative effects of insect herbivores—including generalists and specialists—on the abundance of native competitors relative to the invasive plant. Disproportional effects of generalist deer herbivory on native relative to invasive plants were also found by Knight, Dunn, Smith, Davis, and Kalisz [50] and Kalisz et al. [58]. Moreover, Seabloom, Borer, Martin, and Orrock [51] provided experimental evidence that long-term exclusion of different generalist vertebrates facilitated invasion of exotic species, leading to a decline in native species richness. However, a meta-analysis by Parker et al. [59] showed that native generalist herbivores suppress the abundance of invasive plants, which supports the biotic resistance hypothesis [13]. Thus, depending on the ecological context, either of the two processes can be of major importance [60,61].

There is increasing evidence that belowground enemies can be as important for species co-existence and community dynamics as aboveground enemies [62] and may even drive the positive biodiversity–productivity relationships in native plant communities [63]. Nevertheless, surprisingly few studies have looked on enemy release from belowground enemies [52,64] and most of them concentrated on plant-–soil feedback (e.g., [65,66,67,68,69,70,71,72]. These studies frequently demonstrated that release from soil-borne pathogens may favor plant invasions [52,64], but the role of belowground insect herbivory is still insufficiently understood and studied. Root herbivores are increasingly used as biocontrol agents [73] as they are highly specific and can effectively reduce growth and survival of focal plants species (e.g., [74]. One remarkable outcome of our study was that the presence of *both* above- and belowground insects showed equally strong effects on the establishment of an invasive plant. Besides the release from specialist belowground enemies, the novel chemistry of exotic plants may also act against co-evolutionary naïve insect herbivores feeding belowground [62]. Therefore, current theories on the importance of insect herbivores for plant invasions seem to apply also for the belowground compartment of terrestrial ecosystems [52]. According to theory, invasion success of exotic plants depends on either a competitive advantage over resident species, or on niche differences from the native residents [40]. Our experiment was not designed to disentangle these two mechanisms; however, our results clearly indicate that insect herbivory above- and belowground leads to, or increases, the competitive advantage of invasive goldenrod over its native competitors, thereby contributing to invasion success and impact.

Insect exclusion experiments with the relevant goldenrod taxa (*S. canadensis* s. str. and *S. altissima*) in their native range [4,55] revealed that insect exclusion had a positive effect on population growth rate, leading to a successional pattern of above-ground biomass, which is quite similar to that in Europe without insect exclusion. Thus, whilst insect herbivores exert a direct negative effect on populations of *S. canadensis* in the native range, they indirectly favor the same species in the invaded range via their impact on native competitor species. Hence, an important conclusion from our study is that ecosystem functions are importantly affected by the loss of co-evolved trophic relationships following introduction. The lack of control by specialized enemies and the resulting selective herbivore pressure on native plants results in a competitive advantage of invasive plants in the new range. Moreover, plant invaders are often characterized by increased biomass compared to native plant species [44]. Thus, they have the potential to increase primary production of recipient communities [27]. This leads to the paradox situation that feeding by insect herbivores can increase plant biomass in ecosystems by favoring the establishment of invasive species. Moreover, herbivore-aided invasions lead to the competitive exclusion of native plants and a decrease in plant diversity, which contrasts to the positive effect of herbivores on plant diversity often found in uninvaded communities such as native grasslands [2,3].

## 4. Materials and Methods

### 4.1. Solidago canadensis s. l

Taxonomic affiliation of invasive *S. canadensis* s. l. populations naturalized in Europe is ambiguous: Based on morphological traits, it has been suggested that these populations are taxonomically close to *S. canadensis* var. *scabra* (Muhl.) Tow. & Gray (syn. S. *altissima* L.) despite different chromosome numbers [75]. As these genotypes are different from North American *S. canadensis* L. sensu stricto and are likely to be modified by plant breeding, it has been suggested to term them *S. anthropogena* H. SCHOLZ ined. [76]. Both taxa, *S. canadensis* s. str. and *S. altissima*, are native to North America. *S. canadensis* s. l. was introduced in Europe around 1735, and first naturalized populations were reported from the middle of the 19th century [77]. Today, it is among the most widespread and abundant exotic plant species, and is an aggressive invader of abandoned fields, riverbanks, and reafforestations [75]. Experimental studies show that in the native range, individual growth, clonal, and sexual reproduction [78,79], as well as population dynamics [4,56] of both taxa, *S. canadensis* s. str. and *S. altissima*, are controlled by insect herbivory.

### 4.2. Experimental Design and Treatments

The experiment was conducted on a former arable field with fertile soil (Chernosem, C 1.89%, N 0.16%, NO_3_^−^ 1.09 mg/100 g soil, NH_4_^+^ 0.03, P_2_O_4_^3−^ 46.8 mg/kg soil, K^+^ 176 mg/kg soil) at the UFZ-research station in Bad Lauchstädt near Halle (Central Germany, 110 m a.s.l., mean annual precipitation 490 mm, mean annual temperature 8.8 °C). After the last crop (barley), the field was ploughed in November 1997 and harrowed in February 1998. Thenceforward, the vegetation developed naturally from seeds and root fragments in the soil and from propagules from outside. The insect fauna of this site is characterized by aphids, curculionids, as well as Heteroptera, Diptera, Carabidae, and Hymenoptera [80]. In February 1998, we established 96 experimental plots of 3 m × 3 m. Plots were separated by 2 m wide walkways. We assigned the plots to the following treatments: No insect exclusion (control), aboveground insect exclusion, belowground insect exclusion, and both above- and belowground insect exclusion. The resulting 4 treatments were randomly arranged in 12 blocks with two replicates per block according to a randomized block design.

Herbivory by aboveground insects were reduced by spraying a Perfekthion solution every other week (BASF, dimethoate 40% *w*/*w*; 3 mL diluted in 1 L water, 1 L per plot). Belowground insect herbivory was reduced by applying a Hortex suspension every 4 weeks on the soil surface (Celaflor, chlorpyrifos 2% *w*/*w*, 45 g in 1 L water, 1 L per plot). Plots without insecticide applications were treated with the same amount of water. Depending on the annual climatic conditions, the pesticide treatment began between the end of March or middle of April and ended in October. In an additional treatment, we applied a molluscicide to the half of the plots (Stähler Agrochemie, Limax, metaldehyd 60 g/kg, granulate). However, molluscs were extremely rare on the field site and no side effects of the molluscicide on plant growth could be found [1]. We therefore excluded the molluscicide from the analyses. All pesticides were applied in the recommended concentrations and intervals and are known as effective standard substances for experimental reductions of herbivory (see references in [1]).

### 4.3. Comments on the Use of Insecticides

We are aware that the use of biocides may potentially have direct effects on the vegetation. The two insecticides used in our study have been shown to be very effective insecticides with only weak side effects on non-target organisms ranging from plants to soil microflora [1,81]. Using soil from our experimental field, we could not detect any effects of the pesticides on the growth of the common plant species *Fallopia convolvulus, Chenopodium album*, *Cirsium arvense* [1], and *Solidago canadensis* [82]. However, especially the soil insecticide may also have had effects on non-herbivorous arthropods. Studies with chlorpyrifos demonstrated reduced overall density and shifts in the community composition of springtails in our experimental field [83]. Other authors showed effects of chlorpyrifos on the structure of the springtail community without reducing the overall abundance but no substantial effects on spider communities [84] and oribatid mites [85]. Even if chlorpyrifos in high concentrations may be toxic to earthworms, application at the recommended rates did not show such effects [86]. Fungal and bacterial populations in soil have been shown to be unaffected by the soil insecticide [87,88]. At the recommended concentration and application intervals, the input of nutrients by chlorpyrifos is very low and unlikely to exert direct effects on soil microflora. Eisenhauer et al. [89] and Hector et al. [90] demonstrated only few and rather weak effects of the two insecticides on the germination and seedling growth of different weed species. Thus, they encouraged the use of these pesticides in manipulative experiments on herbivory. We suggest that the use of carefully selected pesticides is a reliable method for assessing the effects of natural insect herbivory on the vegetation and may be even without alternative for soil insects.

### 4.4. Sampling of Vegetation

To assess long-term vegetation dynamics, we used the non-destructive point-square method during the eight years of succession in 10 of the 12 blocks [89]. We placed a sampling frame of 1 m^2^ onto the central square meter of each plot. The frame was regularly divided into 49 quadrats (14.3 cm × 14.3 cm) and a pin of 3 mm diameter was placed randomly within each of these quadrats. We recorded the number of touches by living parts of plant species and used the sum of these touches across the 49 quadrates as an estimate of cover abundance. The sampling was done 8 times per year during the first two years, 5 times per year during year 3 to 5, and one time per year at the biomass peak at the end of June or the beginning of July in the years 6 to 8. For further analyses across years, we used data from the sampling at the biomass peak only.

Old-field successions in the investigation area are characterized by typical sequence of plant functional groups. In the very early years, the community is dominated by annual herbs, which will be replaced by perennial dicots and later on by perennial grasses and woody species. To investigate the effects of treatments on vegetation dynamics, we classified the vegetation into the functional groups *Solidago canadensis*, other perennial herbs (excluding *S. canadensis* and woody species), annual herbs, and perennial grasses. Annual grasses were extremely rare at the study site and therefore excluded.

Immediately after the last point-square sampling at the end of June 2005, aboveground biomass of the vegetation was sampled in the central square meter of all plots. For this, biomass of all species (including *S. canadensis* and resident native species) was clipped at the soil surface, sorted into species, and dried at 50 °C to weight constancy. We calculated species richness as well as Pielou’s evenness index based on Shannon’s diversity index based on biomass data.

### 4.5. Solidago Density

Beginning with the third year of succession, we monitored population density of *S. canadensis* ramets on our plots in September, except in the last year when the final harvest of the experiment constrained us to make the census already in July. On each census date, we counted the number of ramets in the central square meter of plots.

### 4.6. Statistical Analyses

For both the invader and the resident native community, we used cover abundance data (sum of touches per plot) and relative abundances (touches relative to the sum of touches), taken at peak biomass (end of June or the beginning of July), as well as the number of ramets for *S. canadensis* from 2000 to 2005 (years 3 to 8 of the experiment) and analyzed them with repeated measures generalized linear mixed models (Proc Glimmix in SAS 9.0). We excluded the years 1998 and 1999 as *S. canadensis* started to invade in the third year of succession. As first grasses appeared in 2003 only, we excluded all former years in the analysis of this functional group. We used a log-normal distribution for cover abundance, a binomial distribution for relative abundance, and Poisson distribution for the number of *S. canadensis* ramets. A visual inspection revealed that residuals of biomass, species richness, and evenness data were approximately normally distributed, therefore we applied models with Gaussian distribution to these data. In all models, the factor block was considered random and estimated by a restricted maximum-likelihood method.

For the analysis of the community biomass, we only used biomass of herbaceous plant species and excluded biomass of very few tree individuals, which grew on some plot edges since their biomass accumulated over years and did not reflect yearly productivity like in herbaceous plants. However, this did not change the results qualitatively.

## 5. Conclusions

Overall, our experiment clearly demonstrates that release from insect herbivory mediates the invasion success of exotic goldenrod. In addition, it reveals a striking difference in the influences of insect herbivory on ecosystem functions compared to many uninvaded plant communities. This difference provides a mechanistic explanation for the often observed increase in productivity with decreasing diversity in invaded ecosystems. We suppose that the release from coevolved trophic linkages will be of major importance not only for the effect of invasive species on ecosystems, but also for the functioning of novel species assemblages arising from climate change.

## Figures and Tables

**Table 1:** Results from a repeated-measures generalized linear mixed model testing the effect of the duration of the experiment, aboveground insect exclusion, belowground insect exclusion, and the interactions between both factors on the population dynamics (measured as number of ramets, cover abundance) of the invasive plant *Solidago canadensis*. F-values and degrees of freedom are given (significance levels = * *p* < 0.05, ** *p* < 0.01, *** *p* < 0.001).

Fixed Effects	*Solidago canadensis*					
	Number of Ramets			Cover Abundance		
	D.f.	F	Sign.	D.f.	F	Sign.
Time	5, 428	36.5	***	5, 380	83.2	***
Aboveground	1, 81	9.6	**	1, 67	8.1	**
Belowground	1, 81	8.2	n.s.	1, 67	7.0	*
Time x Aboveground	5, 428	0.7	**	5, 380	0.4	n.s.
Time x Belowground	5, 428	0.2	n.s.	5, 380	1.7	n.s.
Above- x Belowground	1, 81	1.4	n.s.	1, 67	0.6	n.s.
Time x above- x Belowground	5, 428	0.6	n.s.	5, 380	0.4	n.s.

**Table 2:** Results from a repeated measures generalized linear mixed model testing the effect of the duration of the experiment, aboveground insect exclusion, belowground insect exclusion, and the interactions between both factors on the community composition based on the relative abundance of the plant groups (*Solidago canadensis,* other perennial herbs excluding *S. canadensis* and woody species, annual herbs, and perennial grasses). F-values and degrees of freedom are given (significance levels = ** *p* < 0.01, *** *p* < 0.001).

Fixed Effects	D.f.	*Vegetation Dynamics*							
		*Solidago canadensis*		Other Perennial Herbs		Annual Herbs		Perennial Grasses	
		F	sign.	F	sign.	F	sign.	F	sign.
Time	5, 380	51.9	***	150	***	11	***	115.3	***
Aboveground	1, 67	20.1	**	7.4	**	17.4	***	9.8	**
Belowground	1, 67	9.3	***	26.2	***	14.2	***	17.4	***
Time x Aboveground	5, 380	0.8	n.s.	1.5	n.s.	0.4	n.s.	1.4	n.s.
Time x Belowground	5, 380	0.5	n.s.	12.3	***	0.5	n.s.	12.2	***
Above- x Belowground	1, 67	1.9	n.s.	0.5	n.s.	10.5	**	<0.1	n.s.
Time x Above- x Belowground	5, 380	1.3	n.s.	0.3	n.s.	0.4	n.s.	0.2	n.s.

**Table 3:** ANOVA results testing the effect of aboveground insect exclusion, belowground insect exclusion, and the interactions between these factors on the diversity and biomass of an old-field community, as well as biomass of *S. canadensis*. F-values and degrees of freedom are given (significance levels = * *p* < 0.05, ** *p* < 0.01, *** *p* < 0.001).

Fixed Effects	D.f.	*Old-field Community*							
		Evenness		Richness		Biomass		*S. canadensis* Biomass	
		F	sign.	F	sign	F	sign.	F	sign.
**Aboveground**	1, 81	7.5	*	4.7	*	11.5	**	19	***
Belowground	1, 81	3	n.s.	0	n.s.	5.5	*	10.1	**
Above- x Belowground	1, 81	3	n.s	0	n.s.	6.1	*	6.8	*
